# Exploring *In Vitro* Mesenchymal Stem Cell Osteodifferentiation *via* Vibrational Microspectroscopy: A Review

**DOI:** 10.1007/s12015-025-10943-3

**Published:** 2025-07-26

**Authors:** Daniela S. Bispo, Inês C. R. Graça, João A. Rodrigues, João T. S. Martins, Mariela M. Nolasco, Maria P. M. Marques, Helena I. S. Nogueira, João F. Mano, Mariana B. Oliveira, Paulo J. A. Ribeiro-Claro, Ana M. Gil

**Affiliations:** 1 Department of Chemistry, CICECO – Aveiro Institute of Materials, Campus Universitário de Santiago, University of Aveiro, 3810-193 Aveiro, Portugal; 2 Department of Chemistry, Molecular Physical-Chemistry - LAQV REQUIMTE, University of Coimbra, 3004-535 Coimbra, Portugal; 3 Department of Life Sciences, University of Coimbra, 3000-456 Coimbra, Portugal

**Keywords:** Mesenchymal stem cells, Osteogenic differentiation, FTIR, Raman, Vibrational spectroscopy, Microscopy

## Abstract

The application of vibrational microspectroscopy to the study of in vitro mesenchymal stem cells (MSC) osteogenic differentiation is a promising approach towards the understanding of the molecular processes involved in bone fabrication. Both infrared (IR) and Raman microspectroscopies have been applied, with a clear predominance of the latter. Bone marrow MSC have been the target of most studies, followed by those originating from dental/oral and adipose tissues. Interests have increasingly addressed single cell and extracellular matrix characterization at the molecular level. Most studies have focused on the characteristics and maturity of time-course mineralization, attempting to localize mineral aggregates formed onto the evolving collagen strands. Some reports have focused on time-dependent changes in protein structure and other components of extracellular matrix components. Besides spectral band examination through position, linewidth and shape, selected band ratios have proved largely informative to assess mineral species evolution and mineral-to-organic matrix interactions over time. The increasing use of multivariate analysis (or chemometrics) and machine learning strategies to detect finer spectral variations is evident, as is the promise of more recent IR and Raman variations to provide higher sensitivity and spatial resolution conditions. The label-free non-invasive nature of vibrational microspectroscopy makes it particularly promising for rapid and effective selection of suitable MSC donors, to support scale-up procedures for translation to the clinic. Some of the challenges to be faced are briefly discussed.

**Graphical Abstract:**

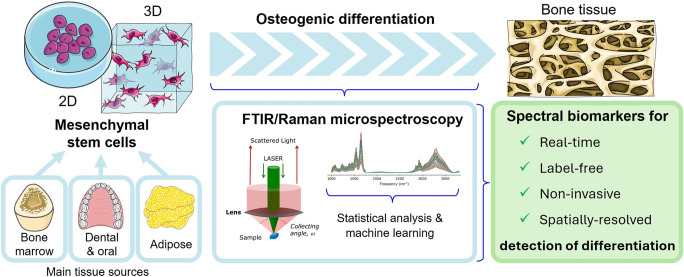

## Introduction

### Mesenchymal Stem Cells (MSC) Osteogenic Differentiation for Bone Regeneration

Natural bone regeneration often struggles to effectively repair large defects and can be further compromised by underlying conditions such as infections or diseases, presenting significant challenges in orthopedics [1, 2]. Current gold-standard treatments, while widely used, come with substantial limitations, including risks of disease transmission (with allografts), donor site morbidity (with autografts), and the need for subsequent surgical removal (with metallic devices) [3]. Moreover, these treatments frequently fall short in terms of efficacy, cost-effectiveness, and biocompatibility. As the global population ages and poor lifestyle choices increasingly compromise bone health [4], there is an urgent need for more effective alternatives in bone tissue engineering (BTE).

Mesenchymal stem cells (MSC) are multipotent progenitor cells capable of differentiating into several cell types, traditionally including osteoblasts, adipocytes, chondrocytes and myocytes. These cells play a vital role in natural bone regeneration and form the basis of many BTE approaches, due to their high ability to differentiate into osteoblasts and secrete bioactive factors [5, 6]. Typically, such strategies combine MSC, either in mono- or co-cultures (or through their secretome/vesicles), with a range of biomaterials and/or physical/chemical cues to enhance the development of functional bone constructs [5, 6]. In this context, MSC are often preferred over induced pluripotent or embryonic stem cells (iPSC and ESC), which, despite their high osteogenic potential, face ethical concerns and a higher risk of tumorigenicity [6]. However, the use of MSC in BTE presents its own challenges. One significant issue is their considerable variability when harvested from different donors, tissues of origin, or even specific cell subpopulations [7, 8]. This heterogeneity, often overlooked in BTE research, remains a significant limitation in achieving standardized and reproducible osteogenic outcomes [5, 7]. While iPSC-derived MSC (iMSC) offer advantages of higher uniformity and differentiation potential, concerns about their tumorigenicity, high cost and complex production processes continue to favor the use of primary MSC in BTE research [9]. Therefore, strategies to understand, monitor and eventually control MSC behavior are essential to optimize the selection of donors and cells with superior osteogenic potential.

**Fig. 1:**
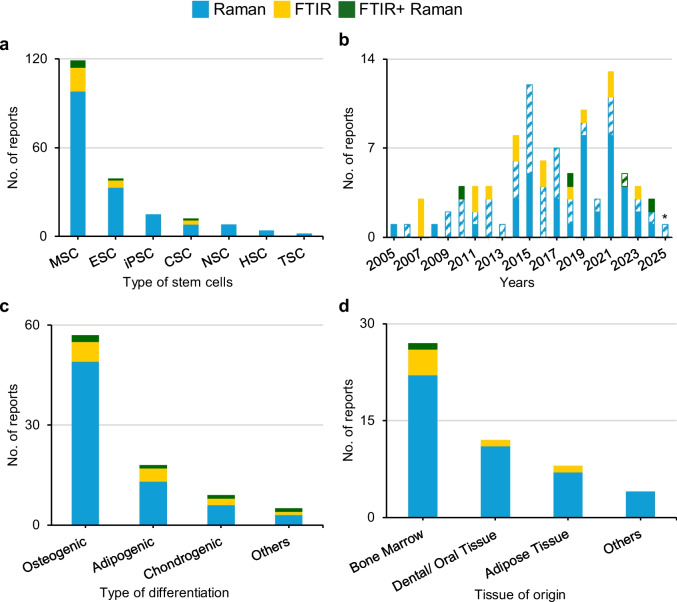
Number of vibrational microspectroscopy reports on stem cells (based on Web of Science database, including papers published up to February 2025). Color code identifies studies employing Raman spectroscopy (blue), FTIR spectroscopy (yellow) and both Raman and FTIR (green). Number of research publications (excluding reviews) are represented as a function of: (**a**) type of stem cells; (**b**) year of publication and status of cells under analysis (live or fixed/dry, in dashed and full bars, respectively), *: up to February 2025; (**c**) type of stem cell differentiation (“Others” includes hepatogenic, osteoclastogenic, fibroblastogenic and myogenic differentiations); and (**d**) tissue of origin of MSC used for osteogenic differentiation. Abbreviations: CSC, cancer stem cells; ESC, embryonic stem cells; HSC, hematopoietic stem cells; iPSC, induced pluripotent stem cells; MSC, mesenchymal stem cells; NSC, neural stem cells; TSC, trophoblast stem cells

The increasing use of non-invasive spectroscopic methods (*e.g.,* nuclear magnetic resonance (NMR), fluorescent-based methods and vibrational spectroscopy) in BTE reflects a growing effort to understand the biology and dynamics of bone regeneration at the molecular level [10–12]. These techniques offer promising label-free strategies for efficiently detecting and optimizing MSC osteocommitment early in the differentiation process [12]. In this context, vibrational microspectroscopy studies have mostly involved Raman spectroscopy, compared to infrared (IR) (in blue and yellow, respectively, Fig. 1a-d), probably due to some usual limitations of the latter (*e.g.,* poor spatial resolution, unavoidable strong water absorption, lack of in-depth resolution), as will be discussed below. This research has mainly addressed MSC (Fig. 1a), often at a single cell level, considering either live or fixed/dried cells (dashed and full bars respectively, Fig. 1b).

Although several differentiation lineages have been explored, a relatively stronger interest has become evident regarding osteogenic differentiation, followed by adipogenic and chondrogenic differentiations (Fig. 1c), mostly studying the behavior of bone marrow MSC (BMSC) (Fig. 1d). The combination of spectroscopic methods with traditional biochemical assays and morphological analysis, throughout osteodifferentiation, has already provided invaluable insight into the nature and dynamics of MSC differentiation into osteoblasts, under different conditions, including increasing information on the ongoing cell/microenvironment crosstalk processes. The growing body of molecular data in this context has not only supported more informed strategies for the development of improved bone tissue-mimicking biomaterials or chemical-free differentiation media, but also unveiled potential reliable biomarkers for the rapid, non-invasive detection of MSC osteogenic differentiation in vitro*.* These biomarkers may help overcome the time-consuming, user-dependent and reproducibility issues associated with current methods of detection of osteogenic differentiation markers [13, 14]*,* while helping to identify optimal donors and suitable osteogenic conditions to facilitate in vitro tissue fabrication for subsequent in vivo implantation and monitoring.

### Vibrational Microspectroscopy in Cell Studies: Principles and Techniques

#### Principles of Vibrational Spectroscopy in a Nutshell

**Fig. 2:**
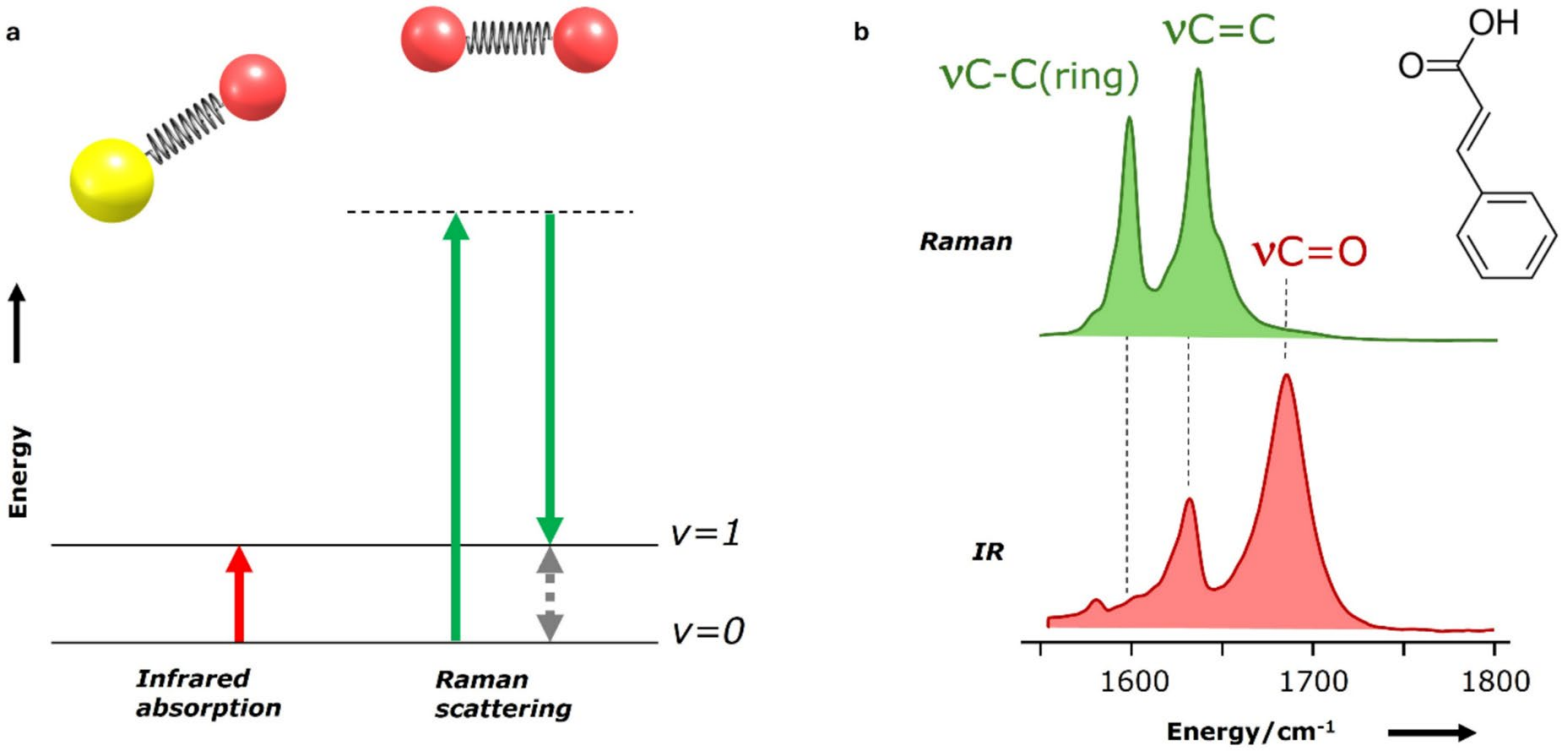
Schematic representation of radiation absorption and scattering processes in molecular vibrational spectroscopy, namely infrared (IR) and Raman spectroscopies. **a**) While IR spectroscopy directly measures the energy difference between vibrational levels (ΔE = E_v=1_-E_v=0_), red arrow), Raman scattering measures the same energy difference in an indirect way. In the latter, the difference between absorbed photons that excite the molecule onto a virtual energy level (dashed line) and scattered photons that bring the molecule to the excited v = 1 level (green arrows) is measured. The intensity of the observed signal depends on the change of dipole moment (for IR) or polarizability (for Raman) associated with the molecular vibration. **b**) Polar bonds tend to yield intense IR absorption bands (bottom), while non-polar bonds tend to produce intense Raman scattering bands (top spectrum), as clearly illustrated by the spectra of cinnamic acid in the region where the stretching vibrations of double bonds absorb

Vibrational spectroscopy probes the vibrational modes of a molecular system, providing information of its structural and conformational features in a highly specific way. The vibrational energy levels depend on various molecular characteristics, including atomic composition, bond strength, geometry, and intermolecular interactions. As a result, vibrational spectra provide exquisitely detailed fingerprints of molecular structure. Two primary techniques, based on the interaction of optical radiation with a sample, enable the study of molecular transitions between different vibrational energy levels: IR spectroscopy and Raman spectroscopy (Fig. 2a). Since most spectrometers no longer rely on a dispersive element (to break incident radiation into its composing wavelengths) and instead use interferometers, the term IR is now replaced by Fourier Transform IR, FTIR. While both FTIR and Raman spectroscopies probe the same vibrational modes, they rely on distinct physical phenomena and therefore follow different selection rules. IR spectroscopy is based on dipole moment variations accompanying molecular vibrations and is particularly effective for detecting polar functional groups such as hydroxyl (-OH), carbonyl (C = O), and amine (-NH). This renders it highly suitable for analyzing biomolecules in cells, particularly in the mid-IR range of wavenumbers (typically from 400 to 4000 cm^−1^) where the most characteristic vibrational modes are detected. In contrast, Raman spectroscopy is based on changes in molecular polarizability associated with each vibrating mode and is particularly sensitive to skeletal modes (including homonuclear vibrations). Raman spectroscopy provides enhanced resolution of features such as the protein Amide III band (a combination of C–N stretching (ν) and N–H bending (δ) vibrations in peptide bonds).

In FTIR this band is usually weak, whereas the Amide I and II modes give rise to intense features, as they arise from the peptide bond ν(C = O) and from a combined δ(N–H) and ν(C–H) mode, respectively. Therefore, it is a commonplace statement that the two techniques are complementary, and this is clearly illustrated for the double bonds stretching vibrations of cinnamic acid, in Fig. 2b. When analyzing biological samples, it is important to note that water is highly absorbing in the mid-IR region which may cause a significant interference in the FTIR spectra [15]. In contrast, water is a weak light scatterer and, thus, it does not hinder the observation of the Raman features of water-rich samples. On the other hand, Raman spectroscopy of biological samples may be hampered by fluorescence due to the presence of intrinsic fluorophores (*e.g.,* aromatic amino acids, heme groups), which can overwhelm the weak Raman signal and mask the spectral features of interest. This aspect needs careful consideration when choosing sample matrixes or substrates on which to present samples for analysis.

Several techniques related to IR and Raman (and associated acronyms) can be found in the literature [16–20], and a complete account of these is out of the scope of this review. However, we hereby highlight some of these, as they are beginning to be explored in cell studies, as explained in the next section. Briefly, for IR spectroscopy, variations are mostly related to the brilliance of the radiation source (*e.g.,* synchrotron radiation, SR-IR, for improved resolution), or the detection method employed (*e.g.,* focal plane array detection, FPA, for imaging). In what concerns Raman spectroscopy, the existing variants usually aim to increase the intensity of the scattered signa, which is the main limitation of the technique. This enhancement may, for instance, be achieved through: i) near-coincidence of the energy of the incident radiation with an electronic transition (resonance Raman, RR, for increasing the signal from a chromophore), ii) interaction with a metal surface (surface-enhanced Raman spectroscopy, SERS, for enhancing the vibrational modes with specific orientations relative to the surface), iii) nonlinear interaction of multiple laser beams (coherent anti-Stokes Raman spectroscopy, CARS, for selective enhancement of a chosen vibrational mode), iv) consideration of the specific polarization characteristics of scattered light (active Raman spectroscopy, ARS, to enhance the Raman signal of specific functional groups).

#### Vibrational Spectroscopy Techniques in Cell Studies

When applying vibrational spectroscopy to the study of cells, both spatial resolution and signal intensity become pivotal. In tandem with optical microscopy, vibrational microspectroscopy becomes a particularly powerful tool for studying heterogeneous samples, such as cells and tissues. In this context, Raman microscopy has emerged as a particularly valuable technique compared to FTIR, offering higher spatial resolution, minimal interference from water (always present in intact cells and tissues), and the capability for in-depth resolution in 3D analysis (with a confocal setup). As the laser beam (exciting radiation) is inherently intense and focused, the spatial resolution of Raman microscopy can be of *ca.* 1 µm within a single xy plane, although a range of 1–200 μm may apply depending on the nature of the sample and wavelength of the exciting radiation. A different situation arises concerning the in-depth or z-axis resolution, which is usually poor in non-confocal Raman microscopy. However, in a confocal set-up, the collected light follows a path through optically conjugated pinhole diaphragms, which provide an efficient ‘spatial filtering’ of the light. In this way, confocal Raman microscopy does typically offer a spatial resolution of *ca.* 1 μm at the surface (xy-resolution) while adding depth resolution (z-resolution) of nearly the same magnitude [16].

The advantage of 3D spatial resolution offered by Raman microspectrosopy is often offset by the intrinsically weak intensity of Raman scattering. Hence, as briefly mentioned before, most of the technological developments of Raman spectroscopy have aimed at improving signal intensity, giving rise to variants such as CARS and SERS although, to the best of our knowledge, these have yet to be widely applied to the study of stem cells. CARS benefits from a highly efficient wave mixing process to enhance the anti-Stokes Raman signal intensity when the energy difference of combined beams coincides with the energy of a Raman active vibrational mode. In this way, CARS provides a label-free, chemically specific contrast with high spatial and temporal resolution, becoming particularly valuable for cell imaging. In addition, the enhancement of the Raman signal of molecules adsorbed onto a metal surface (for instance gold or silver) can be achieved through SERS arising from localized surface plasmon resonance, which amplifies the electromagnetic field, and from chemical interactions between the target molecules and the metal surface. In cell studies, SERS has increasingly employed nanoparticles, either bare or surface-functionalized with biomolecular ligands (*e.g.,* antibodies), for selective binding to cellular components. For a more detailed description of the fundamentals of the above techniques, the authors refer the reader to dedicated reviews, from more general texts [17–20], to several applications in stem cell research, including live stem cell imaging [21, 22].

Compared to Raman microscopy, conventional FTIR microscopy achieves significantly lower spatial resolution, generally limited by diffraction to the wavelength of the exciting radiation (10 – 20 µm). However, since FTIR (relying on digital subtraction of water interference) is a suitable method for studying biological samples such as cells, delivering accurate data on main cellular components, namely lipids, DNA/RNA and proteins, several solutions have emerged to overcome this limitation, enabling micrometer and even nanometer spatial resolution to be achieved. Since conventional IR beams (globar sources) are much larger than the desired spatial resolution, apertures are used to define smaller areas. This approach comes at a cost, since reducing aperture size also decreases the amount of IR light reaching the detector, which in turn limits sensitivity. This loss of sensitivity can be compensated by using high-brilliance beam sources, such as those generated in SR facilities, which also boost the spatial resolution of FTIR microprobes to a few microns [23]. In fact, as stated elsewhere [24], the application of SR can enable spectra with greatly improved spatial resolution and signal-to-noise ratio to be recorded, without the need to resort to prohibitively long acquisition times. In addition, the nanoprobe approach appears as a particularly noteworthy development enabling true absorption-based nanospectroscopy, overcoming the diffraction limit and extends vibrational analysis into the nanoscale (spatial resolution down to 10– 100 nm, *i.e.* well within subcellular scale) [25, 26]. The major advancement of FPA detectors in FTIR microscopy has allowed the simultaneous acquisition of thousands of spectra in a single measurement [27, 28]. In this way, FTIR microscopy can currently achieve a spatial resolution comparable to that of Raman microscopy, enabling highly detailed chemical imaging to be achieved at the subcellular level. The above-described advancements position FTIR microspectroscopy as a cutting-edge technique for the chemical imaging of cells, particularly when leveraging state-of-the-art SR- and FPA-based methods, which push the limits of spatial resolution and analytical capability. To the best of our knowledge, these techniques have yet to be significantly explored in stem cells research.

### Niche and Aims of this Review

This review builds upon previous reports that provided broad overviews of the use of FTIR and Raman microspectroscopies as label-free tools for the characterization of stem cell properties and behavior [12, 29–35]. While these earlier texts importantly focused on general emerging interests and potential advancements in stem cell research, the current review narrows its focus to a detailed examination of primary MSC as progenitors of osteoblasts for BTE applications, a highly relevant topic in modern research.

Some of the prior global reviews have discussed the analytical advantages and challenges associated with different vibrational spectroscopic techniques, alongside the emerging necessity for robust data handling strategies, *e.g.,* statistical multivariate analysis (MVA) (or chemometrics), to detect subtle spectral changes that are not apparent through visual inspection. Briefly, these methods comprise unsupervised MVA methods, such as principal component analysis (PCA) or hierarchical cluster analysis (HCA) [36, 37], to reduce the dimensionality of multiple dimensional complex datasets (as datapoint sets in vibrational spectra) into a representation of a smaller set of dimensions, without requiring prior information on the samples. This captures the sources of data variability, potentially identifying sample clustering due to similarity in their compositional features. Vertex component analysis (VCA) [38] is a less known but significantly promising unsupervised method, which has also begun to be applied to MSC studies by confocal Raman imaging, offering an unprecedented way to visualize submicron-sized intracellular structures, including in 3D cell cultures [39]. Supervised MVA methods, such as linear discriminant analysis (LDA) or partial least squares (PLS) analysis [37, 40] require prior knowledge of sample nature, helping to maximize and interpret group separation and enabling the development of classification models. For a more in-depth discussion on MVA techniques and their application to spectral data, including that obtained through FTIR and Raman spectroscopy, readers are referred elsewhere [37, 41–43].

In the context of vibrational spectroscopy applied to stem cell differentiation, the potential of both unsupervised and supervised MVA methods was initially reviewed for translation of small spectral variations into optical markers of different stem cell populations, end lineages, and stages of maturation and differentiation [29]. These methods were illustrated for murine and human ESC (mESC and hESC, respectively), as well as for some MSC, while also addressing the detection of stem cells within tissues. The need for protocol standardization across laboratories and cross-validation of vibrational spectroscopic findings with established techniques, such as immunohistochemistry, was subsequently emphasized [30]. As mentioned before, Raman spectroscopy has seen increased applicability in stem cell research, outpacing FTIR (blue bars in Fig. 1a-d). An earlier report reviewed Raman spectroscopy applications specifically for neuroprogenitor stem cell differentiation, cardiac differentiation of hESC, and in vitro mineralization of bone nodules [31]. The application of Raman spectroscopy to cell therapies [32] including, but not limited to, stem cell therapies for bone regeneration, was discussed later, identifying a general critical need for effective cell selection strategies for clinical applications. The authors also provided a detailed updated overview of the potential use of several Raman spectroscopy variants, including coherent Raman scattering (CRS) techniques such as CARS and stimulated Raman scattering (SRS), spatially offset Raman spectroscopy (SORS) and SERS. Furthermore, important analytical challenges were discussed*,* including those related to cell fixation and fluorescence interference, as well as the need for sophisticated data preprocessing and adequate statistical analysis.

The most recent reviews have intensified the discussion on the incorporation of machine learning strategies into both FTIR and Raman spectroscopy in stem cell research, while also highlighting the importance of analyzing the extracellular matrix (ECM), the impact of nanomaterials on stem cell behavior (*e.g.,* as drug carriers or targeting agents in SERS), or the characterization of 3D stem cell structures such as organoids and spheroids [12, 33–35]. Regarding the latter, the limitations of live imaging techniques relying on strongly fluorescent probes support a shift toward Raman microspectroscopy, which offers an alternative non-invasive method for accurate 3D structural visualization [12]. The combination of signal-enhancing methods such as CARS, SRS, and SERS with more traditional imaging techniques has been presented as holding particular promise, while full Raman/morphology/biochemical data combination (aided by machine learning methods) has been envisaged as a key strategy for the rapid prediction of differentiation and efficient monitoring of large-scale stem cell production [35].

For readers seeking a broader overview regarding applications of vibrational spectroscopic techniques in stem cell research in general, the above reviews provide a comprehensive foundation. However, as previously noted, the present review will specifically focus on in vitro MSC osteodifferentiation, summarizing the current body of detailed knowledge in this field of bone tissue regeneration.

## Vibrational Microspectroscopy to Study the Osteogenic Differentiation of MSC of Different Origins

Most vibrational microspectroscopy studies investigating MSC osteodifferentiation have employed BMSC (Fig. 1d), likely owing to their higher osteogenic capacity and better-characterized properties, followed by dental/oral MSC (including cells extracted from dental pulp or periodontal ligament tissues, DPMSC and PDLMSC) and adipose tissue MSC (AMSC). Below, we will address these three main sources of MSC separately, followed by a final section on other studies of relevance. For each cell type, the existing FTIR and Raman microspectroscopic applications for characterizing in vitro MSC osteodifferentiation at the molecular level will be discussed, addressing both 2D and 3D cultures, the latter more closely mimicking in vivo bone tissue growth, either biomaterial-based (including scaffolds) or simply cell-based.

### Bone Marrow MSC

#### Bone Marrow MSC Osteodifferentiation in 2D Cell Cultures

An earlier report presented a detailed FTIR microscopy description of human BMSC (hBMSC) osteodifferentiation. The authors considered measurements in several hundreds of single cells [44] and analysis of approximately 260,000 spectra obtained from individual cells. Both unsupervised (cluster analysis) and supervised (LDA) MVA methods were used to compare cells cultured in osteogenic *vs.* growth media. A shoulder in the Amide I band (mainly due to ν(C = O) in peptide bonds), at 1631 cm^−1^, was considered an important marker of undifferentiated cells, suggesting higher contents of *β-*sheet protein structures, compared to osteocommited cells. This was in agreement with higher intensity of the Amide A band at 3285 cm^−1^. Bands in the 1086–1112 cm⁻^1^ region were linked to the formation of calcium phosphate compounds such as octacalcium phosphate (OCP, Ca_8_H_2_(PO_4_)_6_ $\bullet$ 5H_2_O), a precursor to hydroxyapatite (HA, Ca_10_(PO_4_)_6_(OH)_2_). Significant alterations in protein structure also seemed to accompany osteodifferentiation, as evidenced by a decrease in the Amide I shoulder at 1631 cm⁻^1^, with only negligible changes observed in lipids and nucleic acids. Interestingly, two distinct cell subgroups emerged within each of the undifferentiated and differentiated hBMSC groups, highlighting the importance of the intrinsic heterogeneity of MSC, an important challenge in understanding MSC behavior [44]. In particular, two classes of non-stimulated cells were identified as differing in the amount of glycogen deposited in the cell periphery. Also, the maturation stage varied in differentiating cells, with some cells more strongly expressing calcium phosphate compounds (as viewed through the 1086– 1112 cm⁻^1^ region) than others. The authors also highlighted important experimental issues such as the removal of Mie resonance effects, spectral interference of cell matrix in osteogenic conditions, and low signal-to-noise issues.

**Fig. 3:**
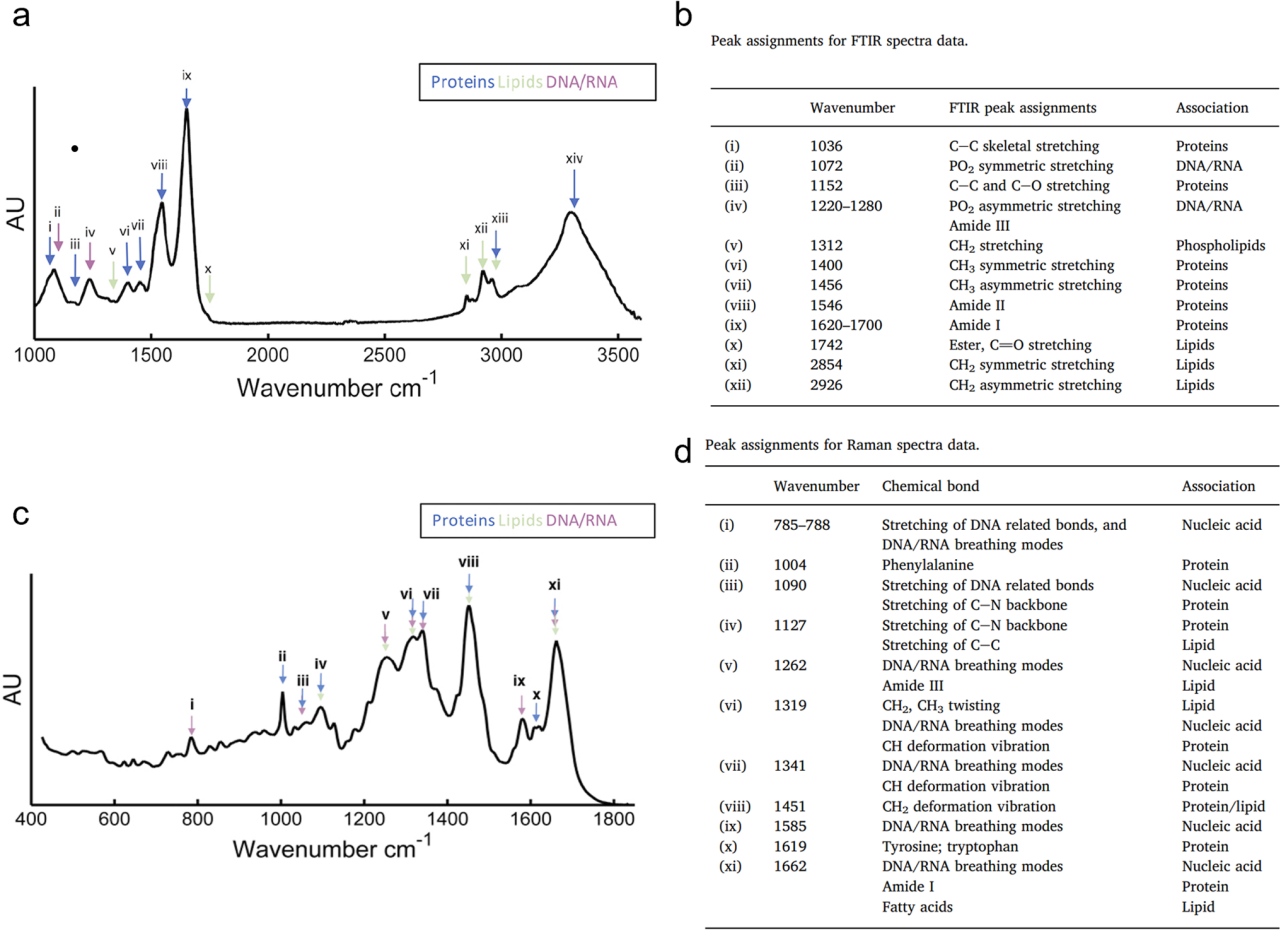
Representative FTIR and Raman spectra of bone marrow mesenchymal stem cells (BMSC), with corresponding assignment. Representative mean (**a**) FTIR spectra with Resonant Mie scattering (RMieS) correction recorded for BMSC with main assignments indicated and listed in top Table, in (**b**), and (**c**) Raman spectra of the same cells with main assignments again listed in bottom Table in (**d**). This figure was adapted from reference [45] 2018, Copyright (2018), with permission from Elsevier

**Fig. 4:**
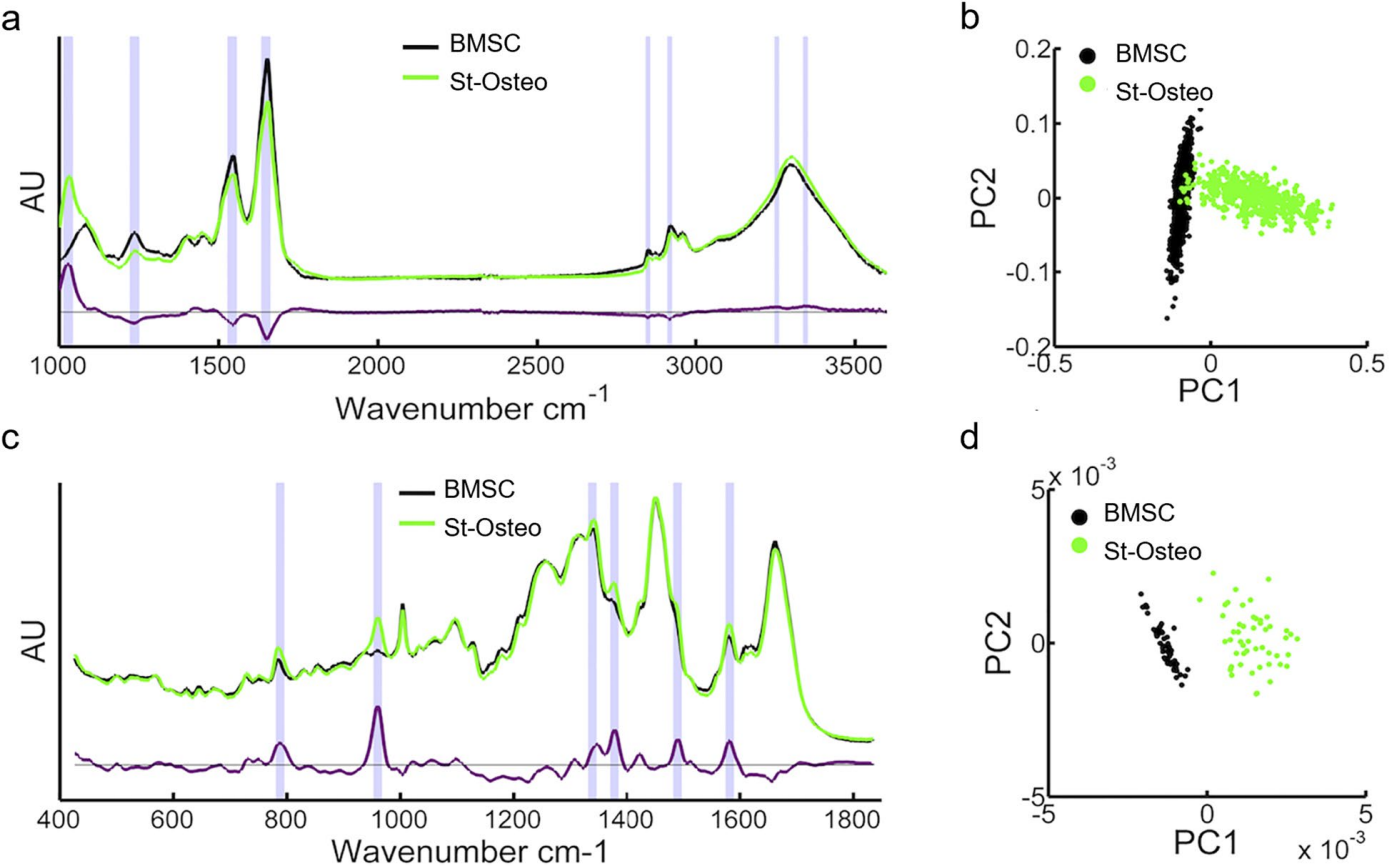
FTIR (**a**, **b**) and Raman (**c**, **d**) spectral analysis of BMSC osteodifferentiation. (**a**) FTIR and (**c**) Raman mean spectra of undifferentiated BMSC (black trace) and corresponding osteodifferentiated MSC (St-Osteo, green trace), together with loading plots of the first principal component (purple bottom trace). (**b**) FTIR and (**d**) Raman PCA score scatter plots comparing undifferentiated BMSC (black circles) and the corresponding St-Osteo (green circles). This figure was adapted from reference [45] Copyright (2018), with permission from Elsevier

Later, FTIR was combined with Raman microspectroscopy to investigate murine BMSC (mBMSC) differentiation [45]. A detailed peak assignment of the FTIR and Raman spectra of undifferentiated mBMSC (Fig. 3a-d), identified bands arising from proteins, lipids and nucleic acids. Aiming to establish a label-free diagnostic method for vascular disease, the authours focused on de-differentiated vascular smooth muscle cells (ddSMC, indicative of disease-related vessel thickening), along with mBMSC and their myogenic and osteogenic progenies. Respectively, these processes mimic natural SMC differentiation (needed for normal vascular function) and exhibit large similarities with vascular calcification. Spectral analysis and MVA confirmed that both FTIR and Raman fingerprints accurately discriminated all cell types under analysis, suggesting these techniques as key to assess lineage purity [45]. Notably, distinguishing between different lineages is a matter of great importance where vibrational spectroscopy may find potential applicability. In this text, the ability of those techniques to differentiate mBMSC from their osteogenic progeny is highlighted. Indeed, the two groups were clearly distinguished by PCA of either FTIR (Fig. 4a,b) or Raman spectra (Fig. 4c,d) and by LDA (built using PCA distinguishing features, or PCA-LDA).

Importantly, the authors validated such models through the corresponding confusion matrixes and classification rates, and results agreed with those obtained through conventional genetic, biochemical and histological data. We note that statistical validation of multivariate analysis models is still seldomly used, despite its utmost importance in translating research results into efficient markers and applications. In terms of spectral profile, osteodifferentiated mBMSC were identified by a strong FTIR feature at 1037 cm^−1^ arising from apatite carbonate groups, overlapped with DNA phosphates at 1070 cm^−1^ (green spectrum and purple trace for PCA loadings (Fig. 4a) corresponding to the scores plot in Fig. 4b). A concomitant intensity decrease in Amides I and III bands was accompanied by changes in the C–H stretching region (*e.g.,* at 2964 cm^−1^) interpreted as arising from both proteins and lipids (Fig. 4a) [45]. The authors reported that Raman spectra of osteodifferentiated cells showed a marked increase in mineral phosphate content, monitored at 960 cm^−1^, and bands associated with carbonate groups (1037 cm^−1^ possibly arising from δ(O–C–O)) and proteoglycans (band at 1375 cm^−1^ for δ(CH₃)) in the ECM. This enabled PCA discrimination of osteodifferentiated cells from BMSC (Fig. 4c,d) and from the remaining types of differentiated cells. The authors acknowledged the complementarity of FTIR and Raman methods for screening and finer discriminant studies of cells [45].

Most studies of BMSC osteodifferentiation have employed Raman microspectroscopy alone, attempting to complement morphological information with additional spectral markers of osteoblast maturity. An initial Raman study used mBMSC cultured gold-coated microscopic glass slides kept in growth medium supplemented with quality elk velvet antler, QEVA. This is a natural extract containing alkaline phosphatase, other proteins, urea and minerals, and known to be involved in the growth and inter-conversion of skin, cartilage and bone [46]. The osteogenic ability of this setup was compared with that of dexamethasone-supplemented and supplement-free media. Raman microscopy characterized different locations of single cells (cell center and filopedia), mainly in relation to HA and several of its precursors [46]. Although the authors also identified Raman bands from other components, namely cholesterol (702 cm^−1^), choline (719 cm^−1^), amino acids and nitrogen bases, the discussion mostly addressed changes in the intensities of HA band at 960 cm^−1^, amorphous calcium phosphate (ACP, Ca_9_(PO_4_)_6_·*x*H_2_O) at 952 cm^−1^, OCP at 957 cm^−1^, and an unspecified HA precursor at 955 cm^−1^, all measured in relation to (or normalized to) the phenylalanine (Phe) band at 1003 cm^−1^, arising from a ring breathing mode, ν_s_(C–C). Notably, in the present context, this Phe band is often considered as a reference band, to enable changes in spectral profile to be accounted for, independently of sample mass or cell number (normalization). The exact protocol for normalization is, however, subject to discussion as shown in some instances below. In the present report regarding QEVA, it was concluded that this extract tended to induce the formation of HA earlier than with the dexamethasone protocol, thus advancing the natural extract as a promising alternative supplement [46].

Subsequently, mBMSC were studied during osteodifferentiation (21 days) through univariate analysis of the average Raman spectra of different cell locations [47]. Selected bands were normalized to the Phe band (1000–1007 cm^−1^), to determine HA/Phe ratios (950–970/1001–1007 cm^−1^, with 950–970 cm^−1^ bands arising from HA ν_s_(${\text{PO}}_{4}^{3-}$)), representing mineral-to-matrix ratios (MTMR). Other Raman spectral features were related to time course evolution of proteins (bands at 1004 and 1650 cm^−1^ from Phe and Amide I, respectively) and carbohydrates (bands at 1450 cm^−1^ for δ(C–H) vibrations). This spectral information was combined with cell morphological data and the authors noted that morphologically longer cells at day 11 constituted the first signs of osteodifferentiation. This was followed by the appearance of bone mineralized nodes between days 14 and 21, reflected by increases at 960 and 1030 cm^−1^ (ν_s_ and asymmetric stretch (ν_as_) ${\text{PO}}_{4}^{3-}$ vibrations), and at 1072 cm^−1^ (ν_s_(${\text{CO}}_{3}^{2-}$)), at day 21. A small shift of the ν_as_(${\text{PO}}_{4}^{3-}$) band to higher wavenumbers was further interpreted as an increase in HA crystallinity at later differentiation stages [47]. Additional work compared bone nodules formation and characteristics resulting from mBMSC and mESC osteodifferentiation with the mineralization of mouse neonatal calvarial (pre-mineralized) osteoblasts (OB) (after 28 days) and native calvarial (skull) mouse bone samples [48]. This work addressed the important issue of the dependence of stem cell osteogenic performance on cell source, employing a multidisciplinary approach, which included standard microscopy and Raman microspectroscopy of live cells. Standard microscopy showed that all cell types led to dense structures, but differed in the dynamics of bone nodules deposition. This was noted to occur more rapidly for mESC. Selected Raman bands (normalized to Phe, 1003 cm^−1^) were analyzed through unsupervised MVA (PCA and factor analysis) [48]. In this way, three different mineral environments were identified based on distinct phosphate bands: crystalline non-substituted HA (962 and 964 cm^−1^), b-type carbonate-substituted apatite (955 and 959 cm^−1^) and amorphous phosphate species (945 and 950 cm^−1^). The band full width at half maximum (FWHM) served as an inverse indicator of crystallinity, along with MTMR values (920–985/1600–1720 cm^−1^ ratios, reflecting ν_s_(${\text{PO}}_{4}^{3-}$) relative to matrix proteins). In addition, carbonate (with ν_s_(${\text{CO}}_{3}^{2-}$) at *ca.* 1070 cm^−1^) to phosphate ratios were also considered. Results showed that OB and mBMSC successfully formed a tissue similar to that of native bone, with type-II collagen serving as intermediary prior to mineralization and later being replaced by a proteinaceous ECM. This matrix seemed to support dynamic mineralization, resembling endochondral ossification [48]. Although osteodifferentiating mESC populations contained a higher proportion of non-osteoblastic cells, they still produced significant amounts of alkaline phosphatase (ALP). This resulted in the rapid formation of mineralized nodules, mostly composed of dystrophic mineral not connected to fibrous proteins and leading to a less stiff material.

Furthermore, the capacity of selected stem/progenitor cells extracted from fetal mouse calvarias (MSC-like cells) to form bone tissue resembling native postnatal calvarial tissue was monitored by Raman microspectroscopy and traditional staining methods, over 28 days [49]. Raman spectra acquired from image locations with suspected mineralization formations enabled the identification of matrix proteoglycans and/or glucosaminoglycans ((CH_3_) in-plane deformation at 1377 cm^−1^). These were expected to be expressed in calvarial tissues and preosteoblasts. Selected band features and ratios gave some indication on cell activity, namely inferred by the nuclei acid band at 782 cm^−1^ (ν(O–P–O) ratioed to either bands from tryptophan (Trp), proline (Pro) or the ω(CH_2_) wag band, at 757, 853 and 1450 cm^−1^, respectively. A decline in cell activity seemed to result from increased matrix synthesis throughout differentiation, while DNA levels were kept constant and cell proliferation downregulated (from around day 8). Mineralization was monitored through the 955–960 cm^−1^ phosphate band, relatively to the Pro band at 853 cm^−1^ (for MTMR). This revealed an interesting bimodal dynamics throughout osteodifferentiation. Also, mineral crystallinity (given by the phosphate band width and position) informed on mineral phases distribution, which depended on culture time. The Amide III subbands at 1246 cm^−1^ (*β*-sheet) and 1264 cm^−1^ (*α*-helix) were related to changes in the relative amounts of disordered to ordered proteins (in days 1 to 4), and on the organization and cross linking of the collagenous matrix (after day 8). Although the authors noted that in vitro tissue was not fully identical to native tissue, the Raman parameters used were advanced as useful to monitor bone quality ex vivo [49].

A PCA-based strategy applied to hBMSC confirmed the evolution of mineralization stages along osteodifferentiation and the involvement of type-II collagen early in the process [50]. This was interpreted as endochondral ossification taking plane before mineralization. Chondrocytes were suggested to later become hypertrophic and give rise to osteoblasts, which are then able to form HA in the ECM. This ECM seemed to be mainly composed by type-I collagen, as shown by the Amides I and III profiles at 1665 and 1245–1270 cm^−1^, respectively, and the ω(CH_2_) wag vibration at 1445 cm^−1^. The authors identified a distribution of different mineral species at days 21 and 28 resembling that of native bone. This seemed to reflect a continuous remodeling of combined older and newer bone tissues [50]. Furthermore, a detailed Raman study of live hBMSC focused on the maturation of the mineralized matrix identifying amorphous calcium phosphate (ACP) (952 cm^−1^), OCP (at 957 cm^−1^), HA (at 960 cm^−1^), *β*-tricalcium phosphate (*β*-TCP, *β*-Ca_3_(PO_4_)_2_, at 970 cm^−1^) and dicalcium phosphate dehydrate (DCPD or brushite, Ca(HPO_4_)·2H_2_O, at 985 cm^−1^) [51]. Analysis of the average Raman spectra recorded on one single cell surface enabled the identification of changes in the bands above, once normalized to the (CH_2_) wag band at 1449 cm^−1^. Notably, the authors experimented normalization to different bands, having found this band preferable to those of Phe or the Amide I. OCP was identified as an important precursor of mineralization, already observable at day 0, *i.e.* in the absence of osteodifferentiation, possibly indicating a potential predisposition of these cells towards osteogenic differentiation. This phosphate mineral form decreased and became undetectable after day 3, being replaced by *β*-TCP from day 9 onwards, along with a marked increase of HA until day 24 [51]. In this study, the contributions of ACP and DCPD were rendered undetectable, and the authors drew attention to more promising Raman variants such as SERS and CARS.

The need for continuous measurements of live cells throughout osteodifferentiation was further emphasized in subsequent reports on mBMSC [52, 53]. The early stages of mineralization (first 3 days in osteogenic medium) were characterized by averaging Raman spectra from three different areas of the bright-field images of live cell cultures. Several bands from non-mineral components were identified (namely, Phe, proteins (Amides I and II), cytochrome c (749, 1127 and 1581 cm^−1^) and carbohydrates δ(C–H) in-plane deformation at 1446 cm^−1^), but emphasis was given to the 958 cm^−1^ HA band to locate mineralization spots. A mineralization map was built based on this band, providing non-invasive microscopic scale information, in complement to the Ca ions distribution given by traditional alizarin red staining [53]. A follow-up study employed time-lapse Raman imaging to follow the mineralization during maturation of mouse osteoblasts obtained by mBMSC osteodifferentiation [52]. Raman imaging was performed every 4 h, up to 24 h, and imaging maps allowed for the detection of HA distribution (956 cm^−1^) to follow mineralization, cytochrome c (750 cm^−1^) for monitoring mitochondrial changes and apoptosis, and *β*-carotene (1526 cm^−1^). Interestingly, the latter was suggested as a marker of mineralization initiation. These observations supported a hypothesis that, during mBMSC osteodifferentiation, *β*-carotene is located near the initiation sites of mineralization where HA precursors accumulate (*e.g.,* of OCP and TCP). The authors advanced that these precursors lead to HA seeding and maturation into hard tissue, while a population of osteoblasts undergoes apoptosis, leaving the surviving osteoblasts to differentiate into osteocytes and/or bone lining cells [52].

The possible competition between osteogenic and adipogenic differentiations is an issue which often arises when using the traditional dexamethasone-based traditional osteoinduction, or which can result from health conditions, such as osteoporosis. In this condition, it is possible that a shift in MSC commitment leads to accumulation of marrow adipocytes during bone loss. This lineage competition was investigated by Raman microspectroscopy, as part of a multidisciplinary strategy, to follow mBMSC differentiation in standard osteogenic or adipogenic media, or in a co-differentiating medium resulting from the addition of 100 nM dexamethasone to osteogenic medium [54]. In the co-differentiation medium, accumulation of both mineralization nodules and lipid droplets (LD) was clearly identified through apatitic calcium phosphate bands (426, 580 and 960 cm^−1^) and several LD bands (between 972 and 1744 cm^−1^). The presence of both osteoblasts and adipocytes was confirmed by complementary methods, showing that the commitment to both lineages was enhanced in the co-differentiating medium, compared to classical media. Raman microspectroscopy was thus advanced as a rapid and non-invasive tool to respond to the challenge of determining co-differentiation and osteoblasts/adipocytes balance. This equilibrium was again addressed to understand how the lipids carried in bone marrow adipocytes impact negatively on mineral density during aging, menopause and even on glycemic control in diabetes type 2 [55]. These authors used Raman spectroscopy to investigate if the ECM initially obtained upon hBMSC adipogenic differentiation influenced subsequent osteogenic differentiation and mineralization quality, once hBMSC were seeded in such ECMs, in dexamethasone-supplemented medium. The effect of different glucose concentrations in both initial adipogenic differentiation and subsequent osteogenic differentiation was also investigated. Raman spectroscopy enabled different ECM components to be identified, namely Pro, hydroproline (HPro) and Phe (fingerprint region, typically < 1500 cm^−1^), Amide I (mainly from collagen), proteoglycans (1064 cm^−1^) and glucosaminoglycans (1318 and 1338 cm^−1^). Spectral changes over 16 days of osteogenesis (normalized to Phe at 1002 cm^−1^) enabled the monitoring of collagen crosslinks (1660/1690 cm^−1^ ratio, a measure of collagen maturity), pyridinolinetrivalent crosslinks (1660/Amide I), mineral to organic ratio (960/Phe). Mineral characteristics were also measured, namely type-b carbonate apatite substitution (as ν_s_(${\text{CO}}_{3}^{2-}$)/ ν_s_(${\text{PO}}_{4}^{3-}$), given by 1070/960 cm^−1^) and HA crystallinity (inverse of FWHM and intensity of ν_s_(${\text{PO}}_{4}^{3-}$)), including size and quality of HA crystals. The authors concluded that the ECM produced by bone marrow adipocytes disrupts the mineral phase of osteoblasts, decreasing cell mineralization and creating abnormal mineralized nodules, with higher number of collagen crosslinks and no b-type carbonate HA substitution [55]. Despite this, no impairment of mBMSC osteocommitment was noted, although glucose concentration in the medium was found to interfere with mineral to organic ratios. This study provided detailed insights into the effect of bone marrow adipocytes on osteodifferentiation, an important step in understanding in vivo function, although care must be taken when translating murine results to human cells.

Another aspect of bone remodeling relates to the need for adequate regulation between osteoclast and osteoblast activities, associated with bone resorption and formation respectively. In this context, a Raman microspectroscopy study, in tandem with PCA and PCA-LDA, investigated mBMSC differentiation into osteoclasts, for 6 days [56]. Most changes occurred after 4 days, mostly in protein bands (Tyr at 851 cm^−1^, Phe at 1002 cm^−1^, Amide III at 1230–1320 cm^−1^, and Amide I at 1656 cm^−1^) and, to a lesser extent, in DNA/RNA and HA phosphates bands at 1095 and 960 cm^−1^, respectively. Such changes seemed indicative of the full maturation of osteoclasts at day 4, after which cell death is believed to begin, involving protein denaturation and conformational changes and degradation of DNA.

#### Bone Marrow MSC Osteodifferentiation in 3D Cell Cultures

A growing interest in BTE strategies involving 3D environments for MSC culture (usually containing biological factors) has led to several applications of vibrational microspectroscopy to elucidate the mechanisms underlying cellular interactions in such conditions. Human BMSC osteodifferentiation was investigated within alginate hydrogel beads containing nacre (mother of pearl, obtained from molluscan shells) powder, a material known to increase cell differentiation and mineralization for bone formation [57]. Structurally, nacre combines calcium carbonate crystals and aragonite (a form of calcium carbonate) within an organic matrix and is detected in Raman spectra through the aragonite vibration at 705–730 cm^−1^. The Raman spectra of alginate bead sections focused on ECM synthesis until day 28. As expected, the presence of nacre powder induced the formation of bone-like HA crystal deposits (detected between 940–975 cm^−1^), but also of its precursor DCPD (or brushite, exhibiting a ν_s_(${\text{PO}}_{4}^{3-}$) band at 986 cm^−1^). Raman mapping based on bands arising from nacre, HA and DCPD indicated that HA forms close to nacre grains whereas DCPD is located at the border of HA deposits.

In addition, two Raman microscopy reports have addressed hBMSC osteodifferentiation within polycaprolactone (PCL) scaffolds [58, 59]. The osteogenic differentiation of these cells was followed continuously for 28 days, exploiting single cell spatial resolution and comparing results with those in 2D cultures [58]. Raman spectra were acquired at the nuclei and cytoplasm and, prior to osteodifferentiation, the spindle-like hBMSC exhibited a spectral profile typical of proteins (skeletal C–C vibrations at 936 cm^−1^, and Amide I and III at 1660 and 1259 cm^−1^) and carbohydrates (in-plane δ(C–H), at 1450 cm^−1^). Osteodifferentiation was evidenced through a change into a cuboidal cell shape and mineralization was detected through 950 cm^−1^ Raman images (ν_s_(${\text{PO}}_{4}^{3-}$) in HA) at day 21. This reflected a considerable delay compared to the expected 1–2 weeks in 2D cultures. Interestingly, Raman images guided by the 2900 cm^−1^ ν(C–H) band, suggested the concomitant formation of LD, although their visualization was somewhat compromised by overlap with a PCL band [58]. This expresses the importance of selecting Raman-transparent substrates, as far as possible. PCL was also used to produce a material replicating nacre topography, intending to show that the osteoinductive properties of native nacre may, at least in part, be due to its surface patterning [59]. The osteodifferentiation of hBMSC on PCL nacre-replica surfaces was compared with that achieved on topographic surfaces (namely, a surface with a near square disordered (NSq50) pattern and nacre shell, the latter serving as a combined topographic/chemical cue), and in 2D cultures in osteogenic media (supplemented with either dexamethasone or cholesterol sulfate). The authors presented a detailed Raman assignment of the cellular matrix, including identification of proteoglycans (1060 cm^−1^), phospholipids (1127 cm^−1^) and glycosaminoglycans, overlapped with other proteins and lipids signals in the fingerprint region. As expected, osteodifferentiation changed the characteristics of the ν_s_(${\text{PO}}_{4}^{3-}$) band and MTMR values (given by ν_s_(${\text{PO}}_{4}^{3-}$)/HPro at 853 cm^−1^). The results showed that nacre topography alone does result in distinct nascent mineral forms and more mature HA, with crystallinity and MTMR indicative of better-quality bone tissue [59]. Interestingly, the authors also showed, through liquid chromatography-mass spectrometry (LC–MS) metabolomics, that different topographic nuances impact more strongly on cell metabolic phenotype/function than different chemical methods, reflecting the high sensitivity of cell activity to biophysical cues [59].

In the context of bioreactors for bone tissue fabrication in vitro, non-invasive and continuous characterization of the osteodifferentiation of live BMSC cells has been carried out by Raman microspectroscopy [60–62]. Initially, two microbioreactors were developed for optical coupling to a Raman microspectrometer, with different geometries to serve distinct purposes: i) characterize the molecular level changes during osteodifferentiation and ii) enable chemical imaging of organelle distribution within cells [60]. To avoid strong Raman scattering from the organic polymers, the continuously perfused microbioreactors were made of Raman transparent materials (namely, CaF_2_ and borosilicate glass). At different time points, cells were collected and their Raman spectra analyzed by unsupervised MVA (HCA). The expected increase of the phosphate band at 961 cm^−1^ on day 21 was indicative of HA formation, while the absence of bands at 1578 and 1607 cm^−1^, interpreted as reflecting necrotic cells, suggested that hBMSC remained metabolically active. HCA of Raman spectra provided images, which enabled the identification of plasmatic and organelle membranes (through the phosphatidylcholine band at 718 cm^−1^), DNA distribution in the nuclei (through nucleotide bands at 729–1575 cm^−1^), intracellular lipids (718–1740 cm^−1^) and even mitochondria (small band at 1602 cm^−1^). Hence, the authors convey that microbioreactors combined with Raman microspectroscopy are a valuable tool to study MSC proliferation, differentiation, and development into tissues under in situ and in vitro conditions [60]. A subsequent report advanced a bioreactor focused on the mineralization process of osteodifferentiating MSC (of an unclear origin) [61], where cell aggregates started to form at day 12 and increased in size thereafter. Changes in the ν_s_(${\text{PO}}_{4}^{3-}$) band (position, intensity and shape) indicated temporal changes in mineral phases, with PCA and Raman spectra deconvolution revealing mineral aggregates of different sizes and shapes (specifically including formations of *ca.* 40 μm). Considering the phosphate bands at 958 cm^−1^ and 948 cm^−1^ as indicative of crystalline and amorphous HA phases respectively, results showed that the crystalline phase increased with time in each nodule, with the amorphous phase positioned at the edges.

More recently, a Raman microspectroscopic study of hBMSC osteodifferentiation involved a 3-component bioreactor environment derived from chitosan, agarose and HA nanopowder, having compared static, perfusion and rotating conditions [62]. The rotating bioreactor resulted in the production of higher amounts of osteopontin (OPN), a marker of osteodifferentiation, with Raman spectra showing enhanced ECM mineralization through b-type carbonated substitution of HA and higher MTMR values. PCA performed with 400 spectra per bioreactor distinguished perfusion and rotating bioreactors, mainly due to impact on phosphate bands in the 1012–1096 cm^−1^ region. Overall, the rotating bioreactor induced a superior effect in cell proliferation, ECM synthesis and mineralization, giving rise to a clearer bone-like structure. Interestingly, the extent of osteodifferentiation (upon normalization by cell number) did not seem to differ between static and rotating conditions, although cell growth distribution were significantly distinct (only on the biomaterial top in static cultures and over the entire surface in dynamic conditions).

### Dental/oral MSC

#### Dental/Oral MSC Osteodifferentiation in 2D Cell Cultures

**Fig. 5:**
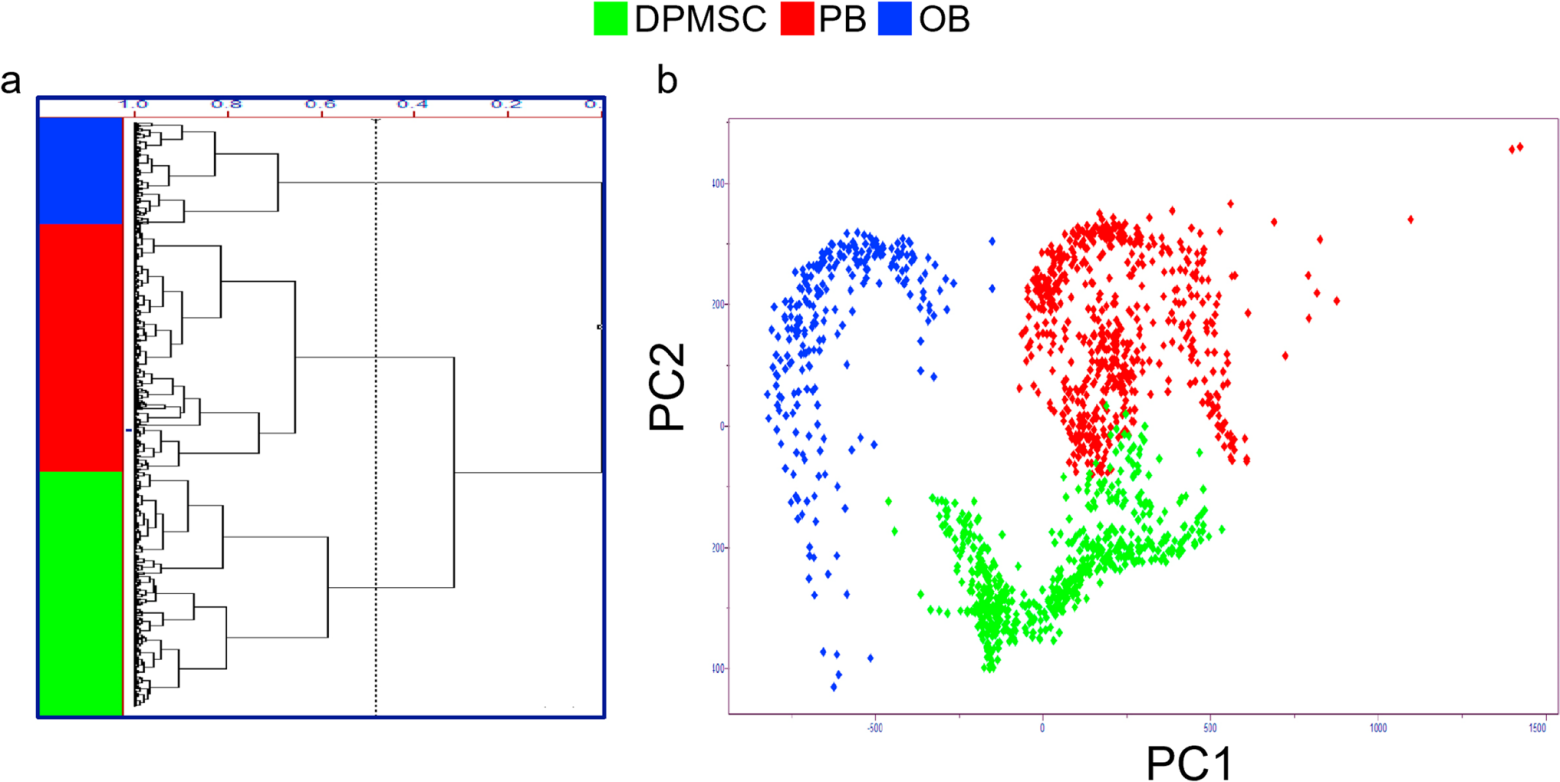
Unsupervised multivariate analysis of FTIR spectra collected throughout the osteodifferentiation of dental pulp MSC (DPMSC). (**a**) HCA analysis and (**b**) PCA scores plot comparing undifferentiated cells (green), preosteoblasts (PB, red) and osteoblasts (OB, blue) in the 1800–900 cm^−1^ range. Adapted from reference [63]. Copyright (2011), with permission from Elsevier

Dental pulp MSC (DPMSC) are obtainable in a relatively easy way from young patients, however, to our knowledge, only one FTIR microscopy account has been reported in the context of the osteodifferentiation of this cell type [63]. In such study, the authors compared the FTIR spectra of undifferentiated DPMSC cells, preosteoblasts (at day 10) and osteoblasts (at day 20), having noted that the main spectral changes affected the Amides I, II and III bands and phosphate stretching vibrations. With the aid of unsupervised MVA of vector normalized spectra (namely PCA and HCA) three clusters were identified corresponding to the different cell groups (Fig. 5a,b). Second derivative spectra and peak deconvolution in the 1470–1720 cm^−1^ region indicated an enhancement of *α-*helical features at 1653 cm^−1^ and Amides I, II and III (at 1665, 1548 cm^−1^ and 1314/1290 cm^−1^, respectively), compared to those typical of *β*-sheet (intermolecular at 1696 and 1612 cm^−1^, and intramolecular at 1681 and 1630 cm^−1^) and random coil conformations (at 1643 cm^−1^). These changes were interpreted as reflecting a rise in the osteogenic markers type-I collagen and osteocalcin (OCN) from day 10 to day 20, consistent with the predominantly helical structures of these proteins [63]. Furthermore, while the authors did not discuss clear evidence of mineralization, changes in phosphate vibrations were identified through the FTIR ν_s_(${\text{PO}}_{4}^{3-}$) band at 1236 cm^−1^ assigned to nucleic acids phosphodiester bonds and suggested to adopt an intermediate hydration state between hydrogen-bonded and non-bonded in osteoblasts.

In relation to Raman studies of DPMSC, one first account considered their osteodifferentiation by mainly focusing on the ν(C–H) region (2800–3000 cm^−1^) and the ν_s_(${\text{PO}}_{4}^{3-}$) band at 960 cm^−1^ [64]. On day 21, the latter band became clearer, together with other phosphate bands (${\text{PO}}_{4}^{3-}$ deformation modes at 430 and 585 cm^−1^). Raman images were obtained for the ν_s_(${\text{PO}}_{4}^{3-}$) vibration and ν(C–H) region, and the ratio of these images was computed to obtain 960/2800–3000 cm^−1^ maps, which enabled the identification of mineral phosphate around cells. Other bands identified arose from Phe at 1006 cm^−1^, δ(CH_2_) at 1460 cm^−1^, ν(C–C) at 1609 cm^−1^, ν(C = C) of lipids overlapped with the Amide I at 1654 cm^−1^, and CH_3_ ν(C–H) at 2887 cm^−1^. Notably, collagen (detected at 1032 cm^−1^) seemed absent inside the cells and localized around them, similarly to phosphate. Later, the same authors focused on the process of ECM maturation during DPMSC osteodifferentiation [65], revealing interesting top and lateral sample views. Raman maps again explored the distribution of the 2800–3000 cm^−1^ region (as a measure of all cells), the 960 cm^−1^ ν_s_(${\text{PO}}_{4}^{3-}$) band and mineral to organic ratios across images, as well as the 1660/1690 cm^−1^ ratio (Amide I subbands) to measure the extension of collagen cross linking. This was related to collagen maturity and bone stiffness, namely to trivalent cross-linked pyridinoline and divalent cross-linked dihydroxylysinonorleucine. Raman images reflecting this ratio indicated that collagen cross linking appeared after day 14 in osteogenic medium and was delayed in the absence of such stimulus.

In another study, a quartz window customized flask was prepared for rapid Raman spectroscopy of live cells in aseptic conditions, with the authors usefully outlining a clear stepwise protocol for DPMSC osteodifferentiation [66]. Cells from three donors were considered and PCA was employed on averaged spectra, having revealed an interesting two-staged process throughout osteodifferentiation: a protein increase until day 10 (1453 and 1660 cm^−1^), followed by a relative DNA/RNA decrease accompanied by the expected increase at 955–964 cm^−1^ due to mineralization.

Furthermore, to investigate how the tissue of origin of dental/oral MSC impacted on the characteristics of the end tissue, a Raman study characterized the mineralized nodules originating from six types of cells isolated from human teeth and supporting tissues (bone chip mass population, BCMP; dental pulp adult, DPA; gingival fibroblast, GF; stem cells from apical papilla, SCAP; periodontal ligament, PDL; and stem cells from human-exfoliated deciduous teeth, SHED) [67]. After 28 days of osteogenic induction, nodule composition was compared to dentine, enamel and cementum, considering selected spectral areas and positions (normalized to unit area and upon curve fitting), FWHM and MTMR values (given by 960/1660 cm^−1^, ν_s_(${\text{PO}}_{4}^{3-}$)/Amide I). In addition, PCA was carried out on the average spectra of each group of samples. For all sample groups, mineral deposits were detected on day 28 as confirmed by histochemical staining, but Raman spectroscopy added more detail on the mineralized matrix. In particular, all end tissues contained a carbonate-substituted apatite-like mineral on an organic matrix. This was consistent with all materials showing similarities to dentine/enamel, while none matched native dental tissue. However, deposition patterns and MTMR values were dependent on cell type, demonstrating the importance and need of correlating different MSC origins with end outcomes [67].

The already mentioned important issue of distinguishing the osteogenic lineage from possible competing co-lineages was investigated by Raman microspectroscopy of human periodontal ligament MSC (hPDLMSC), upon stimulation to differentiation into osteogenic, adipogenic or chondrogenic lineages, as assessed by traditional methods (namely alizarin red and lipid oil red O staining, and safranin O testing for cartilage-specific proteoglycans) [68]. For all conditions, the differentiation status was assessed by PCA of Raman spectra, after spectral subtraction of the glass substrates signal (chosen for their availability and low cost), normalization to the 1660 cm^−1^ band (chosen for its high intensity) and scaling. Considering a detailed spectral assignment of bands arising from amino acids, proteins and nucleic acids, the authors corroborated previous suggestions that the concomitant increase of proteins/lipids bands and decrease of nucleic acid bands may help distinguish more mature cells. Furthermore, although the issue of heterogeneity between different hPDLMSC batches was shown to play a role, it still allowed for the fast and simple identification of the different lineages, based on the corresponding Raman spectral profiles [68].

#### Dental/Oral MSC Osteodifferentiation in 3D Cell Cultures

The deposition of bone nodules arising from osteodifferentiating gingival MSC on titanium surfaces was studied for 28 days and compared to native alveolar bone, using scanning electron microscopy (SEM) for morphology characterization and Raman microspectroscopy [69]. Band intensities at 960 (ν_s_(${\text{PO}}_{4}^{3-}$)), 1667 (Amide I), 1245 (Amide III), 1451 (δ(CH_2_)) and 2800–3100 cm^−1^ (ν(C–H)) were found to be similar to those of native alveolar bone. However, the authors highlighted the need to further investigate the effects of different variants of the implant surfaces (*e.g.*, oxidized or sandblasted). A more recent paper addressed a comparison of osteodifferentiating hPDLMSC in scaffold-free 3D clumps of cells, compared to 2D cultures, with particular focus on the effect of lipopolysaccharides (LPS) on the process [70]. This was justified by the presence of LPS in the outer membrane of gram-negative bacteria linked to chronic periodontal disease, and the possible interference of those polymers with hPDLMSC osteogenic potential. Raman microspectroscopy showed that, in 2D cultures, LPS induced changes at 960 (ν_s_(${\text{PO}}_{4}^{3-}$)), 785 (ring-breathing modes of DNA/RNA bases) and 855 cm^−1^ (Pro and HPro). Together with other data (namely alizarin red and ATP-based proliferation assays), these changes were interpreted as reflecting decreased osteogenic potential and proliferation. Interestingly, however, in 3D clumps cultures, the osteogenic potential of hPDLMSC seemed to be retained. This suggested a higher resistance of 3D cell aggregates towards the cytotoxic effects of LPS, thus supporting their use in scaffold-free therapeutic strategies.

### Adipose tissue MSC

#### Adipose Tissue MSC Osteodifferentiation in 2D Cell Cultures

**Fig. 6:**
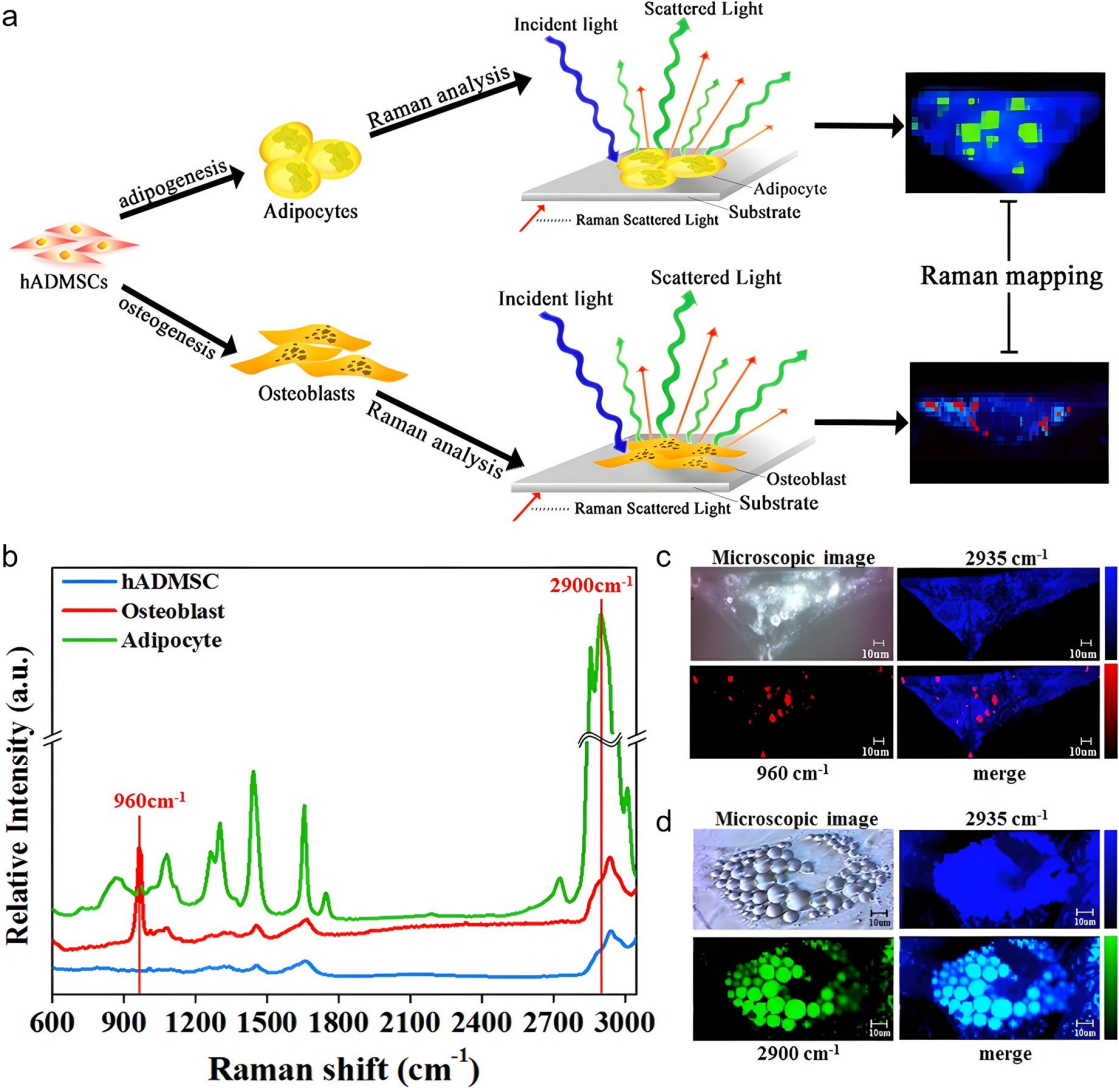
Raman spectroscopy workflow and analysis of hAMSC differentiation. (**a**) Schematic workflow of Raman spectra acquisition for adipogenic and osteogenic differentiation of hAMSC. (**b**) Average Raman spectra (single cell) from hAMSCs (blue), osteoblast (red) and adipocyte (green). (**c**) Microscopic and Raman images relative to CH_3_ νC-H (2935 cm^−1^) and phosphate ν_s_ vibration (960 cm^−1^) bands, for unstained osteoblasts. (**d**) Microscopic and Raman images relative to CH_3_ νC-H (2935 cm^−1^) and lipid νC-H (2900 cm^−1^) map, for unstained adipocytes. Adapted from [71], Copyright (2018), with permission from Elsevier

To the best of our knowledge, a first investigation of adipose tissue MSC (AMSC) osteogenic differentiation by vibrational spectroscopy employed Raman microspectroscopy and addressed live human AMSC (hAMSC), either exposed to osteogenic or adipogenic media for 21 days (Fig. 6a) [71]. Measurements were taken every 3 days using single cell Raman spectroscopy with an immersion lens. The authors presented a useful discussion of the best choices of substrates (tissue culture plate, slide glass, silicate glass and quartz) and instrumental conditions, having selected quartz and a 10 μm z-axis distance for the best cell-specific Raman imaging. The analysis was strongly based on Raman mapping guided by the bands at 960 cm^−1^ (HA), 2900 (lipids in cytosolic LD) and 2935 cm^−1^ (ν(C–H) from organic methyl groups) (Fig. 6b) and the 960/2935 cm^−1^and 2900/2935 cm^−1^ratios. The 2935 cm^−1^ band was the most intense in the spectra and, as it arises from organic methyl stretching vibrations in general, it was used to visualize cell shape (Fig. 6c,d). The HA and LD bands were found to be highly specific of osteoblasts and adipocytes respectively, having provided clear Raman images of the dynamics of formation of each lineage [71]. In this way, osteodifferentiation was detected 9 days after induction (*i.e.*, one week earlier than when detected with the traditional alizarin staining method), whereas the increase of cytosolic LD in adipogenic single cells was detected during the entire 21-day period. As a result, the method was strongly advised to support precise *in-situ* monitoring of AMSC differentiation.

#### Adipose Tissue MSC Osteodifferentiation in 3D Cell Cultures

Notably, most of the AMSC studies in the context of osteogenic differentiation have, to our knowledge, involved cell cultures in scaffolds. A recent FTIR report characterized the processes of hAMSC osteodifferentiation in comparison with that of hBMSC, within a hybrid scaffold composed of chitosan, *β*-1,3-glycan and HA, using macro attenuated total reflectance (ATR)-FTIR imaging [72]. The authors focused primarily on human AMSC given their easy accessibility and fast-paced proliferation, while exhibiting comparable morphology, phenotype and in vitro differentiation abilities to human BMSC. The study focused on the efficiency of the scaffold to support MSC osteodifferentiation, through the measurement of collagen molecular structure and maturity, and of mineral formation and crystallization. Specific IR bands were selected and normalized (mostly to the C–O skeletal band at 1027 cm^−1^, or to the whole Amide I, II and III bands region) and used to compute particular band ratios. This information was used to monitor: i) secondary protein structure and collagen-fibril orientation (through Amide I/Amide II ratios); ii) collagen cross-links and maturity (1660/1690 cm^−1^, reflecting mature/immature collagen); iii) mineral maturity and crystallinity (1030/1100 cm^−1^); iv) MTMR values (through ν_s_(${\text{PO}}_{4}^{3-}$)/Amide I); and iv) carbonate modification of HA (ν(C–O)/Amide I). Macro ATR-FTIR images (of 0.6 × 0.55 mm) were based on bands arising from lipids, proteins, carbohydrates and phosphates and enabled the identification of scaffold spectral contributions and hAMSC and hBMSC ostoeodifferentiation comparison [72]. Both MSC types produced high amounts of type-I collagen (corroborated by confocal microscopy) and comparable collagen maturity ratios. However, hAMSC led to higher collagen quantities, with a predominating *β*-turn structure, as opposed to the main triple-helix structure detected in hBMSC-derived collagen. As the most abundant protein in ECM, collagen provides ductility, energy-absorbing capability and stiffness to fresh bone tissue and the impact of such molecular characteristics on ECM quality was discussed. Despite some slight compositional and structural.

differences, unsupervised multivariate analysis (namely, HCA) and spectral deconvolution showed that the two MSC types performed similarly, both types being able to alter lipid profiles, known to impact cell physiology, influence initiation of collagen fiber mineralization, and even trigger scaffold modifications (namely affecting HA carbonate and phosphate contents). The ability of ATR-FTIR to circumvent the non-specificity of staining techniques on scaffold-based studies was highlighted [72]. The same authors subsequently reported a Raman spectroscopic imaging comparative study of the same cell types [73]. The expression of proteins in the ECM was confirmed on the scaffold surface for both types of MSC (through measurement of Amides I and II bands), as well as adsorption of media proteins on the biomaterial surface evidencing high biocompatibility of the scaffold. Raman mapping was also employed to characterize the arrangement of the function of the different scaffold components. Interestingly, HCA results were consistent with higher HA contents arising from hAMSC, compared to hBMSC. Analysis of HA structure and crystallinity, aided by Raman mapping based on the 961 cm^−1^ band, illustrated the formation of a new HA layer in hAMSC-seeded samples, demonstrating surface biomineralisation of the scaffold.

As also noted above for BMSC, most reports on AMSC osteodifferentiation processes have employed Raman microspectroscopy. In particular, the increase of the Raman band at 960 cm^−1^ (normalized to the average intensity between 475–560 cm^−1^) was used to monitor the extent of HA formation by hAMSC encapsulated in gellan gum/type-I collagen hydrogels during osteodifferentiating induced by bioactive glass ions [74]. Subsequently, a more detailed study compared hAMSC osteodifferentiation in 2D cultures with that in 3D structures obtained by adding demineralized bone matrix as the scaffold [10]. An impressive number of hAMSC batches (*n* = 21) from 10 different donors was considered and biopsies of the 3D forming bone tissue were regularly extracted until day 56. The consideration of multiple donors and batches is a strength of this study, as the impact of these issues on MSC performance are still far from understood. Furthermore, the specificity of molecular changes to osteodifferentiation was evaluated by comparison with a similar protocol for the induction of chondrogenic differentiation, which is particularly pertinent given the shared biological characteristics of these lineages, especially during endochondral ossification. Harvested biopsies were compressed between 2 quartz disks and Raman images recorded considering bands at 960 (HA), 1002 (Phe) and 878 cm^−1^ (ν(C–C) from HPro), as well as the 960/1002 cm^−1^ and 960/878 cm^−1^ ratios, interpreted as MTMR values. These ratios indicated delayed mineralization in 3D structures (start at day 28), compared to 2D cultures (maximum reached at day 14), similarly to a previously mentioned 2D/3D comparative study of BMSC osteogenic differentiation [58]. Importantly, the repeatability and specificity (towards chondrogenesis) of the 3D demineralized bone scaffold were quantified through both Raman maps and PCA of the average Raman spectra. The results showed that MTMR values in 3D biopsies peaked at days 35–49, although with a large donor dependence. This consideration of donor heterogeneity is of utmost importance for such time dependence to be considered as guidance for use of bone grafts for patient surgery. Furthermore, the authors observed that a maximum MTMR of 4 (based on Raman band measurements) was advised for implantation, and that spatial dependence of mineralization (as assessed by Raman) also needs to be addressed.

More recent reports have addressed AMSC in alginate-based scaffolds [75, 76], in the presence of natural components believed to have the ability to boost osteogenic differentiation. In one study, the phytochemical thymoquinone (TQ) was added to an alginate-impregnated HA scaffold and compared to traditional osteogenic conditions (dexamethasone-based medium), TQ with dexamethasone-based medium and, finally, TQ alone (in a dexamethasone-free medium) [75]. The latter condition expressed the current challenge of finding alternatives to the traditional dexamethasone-based induction protocol. Raman spectroscopy used to follow mineralization and impact on scaffold HA (500–1000 cm^−1^) and on organic scaffold components (1000–2000 cm^−1^) revealed that, in the presence of osteogenic medium, TQ seems to accelerate differentiation. This observation prompted further analysis of TQ impact on scaffold mechanical properties, and of TQ release and toxicity properties. A similar investigation was carried out using Shilajit (or mineral pitch), a herbomineral natural substance used to treat bone defects in Iranian folk medicine and shown to correspond to a complex polysaccharide-like ^1^H NMR spectral profile [76]. The addition of Shilajit to a 3D alginate hydrogel (also compared to 2D cultures) was analyzed through Raman spectroscopy in different scaffold regions, considering spectral changes at 500–2000 cm^−1^ to monitor mineralization (HA at 966 cm^−1^ and calcium carbonate in apatite at 1073 cm^−1^) and type-I collagen formation (820 cm^−1^). In addition, Raman spectra identified free and esterified cholesterol, monosaccharides, disaccharides and polysaccharides (amylose and amylopectin), carotenoids and several aromatic structures) in Shilajit. Most importantly, Shilajit was found not only to display osteogenic properties, but also to boost differentiation in osteogenic medium, without changing the alginate hydrogel physical properties. The authors discussed the release properties of Shilajit components, their biodegradability and antioxidant properties, the latter suggested to potentially inhibit bone loss and promote bone repair.

### Other Studies

This section puts together vibrational microspectroscopy reports of MSC from other (or undefined) tissue sources, the study of which brings significant novelty in terms of technological advancements or biochemical insights. As noted earlier, within the current developments in FTIR microspectroscopy, SR-FTIR has been increasingly revealed as an effective method for studying cell characteristics. This has been illustrated in a study of differentiating umbilical cord MSC (UCMSC), not only into osteoblasts but also into adipocytes since, as mentioned before, an adequate balance between the two lineages is determinant of osteogenic outcome [77]. Such balance can be achieved by pharmaceutical or genetic modulation of an effector protein named Yes-associated protein (YAP) and, indeed, higher YAP expression levels resulted in enhanced osteogenic differentiation compared to adipogenic (whereas a reversal was noted for lower YAP expression levels). This was supported by unique SR-FTIR fingerprints found for YAP-targeted differentiated cells, clearly distinguishable from non-manipulated controls, and highlighting specific bands from lipids (C–H stretching region), proteins (Amides I and II), nucleic acids, carbohydrates and phosphate. In addition, PCA of more than 200 FTIR spectra of single cells discriminated 3 clusters corresponding to controls and cells with overexpressed or knocked down YAP, identifying more intense ${\text{PO}}_{4}^{3-}$ bands at 977 and 1000 cm^−1^ and of the nuclei acids phosphodiester band at 1226 cm^−1^ for cells with overexpressed YAP. These results seemed to demonstrate the role of YAP in promoting UCMSC osteodifferentiation and the authors highlighted the role of SR-FTIR to provide highly sensitive chemical fingerprints of differentiating singe cells. More recently, single cell SR-FTIR was also applied to polyvinyl alcohol (PVA) hydrogels loaded either with lithium chloride or with the Wnt5a protein to test the osteogenic induction capacity of these agents towards human MSC (hMSC) [78]. Exogeneous lithium chloride and the Wnr5a protein are believed to share a similar function in the activation of the Wnt signaling pathway in cell differentiation and their loading in PVA scaffolds aimed at investigating the exogenous agent-PVA synergetic impact on hMSC osteodifferentiation. Again, PCA of SR-FTIR spectra was used, this time adding calculated cell–cell Euclidean distances to evaluate cell heterogeneity during osteogenic differentiation. This demonstrated that the use of PVA hydrogel can lead to distinct effects of the exogeneous agent, highlighting the importance of niche function in hMSC osteodifferentiation and SR-FTIR as a rapid effective tool to address it.

**Fig. 7:**
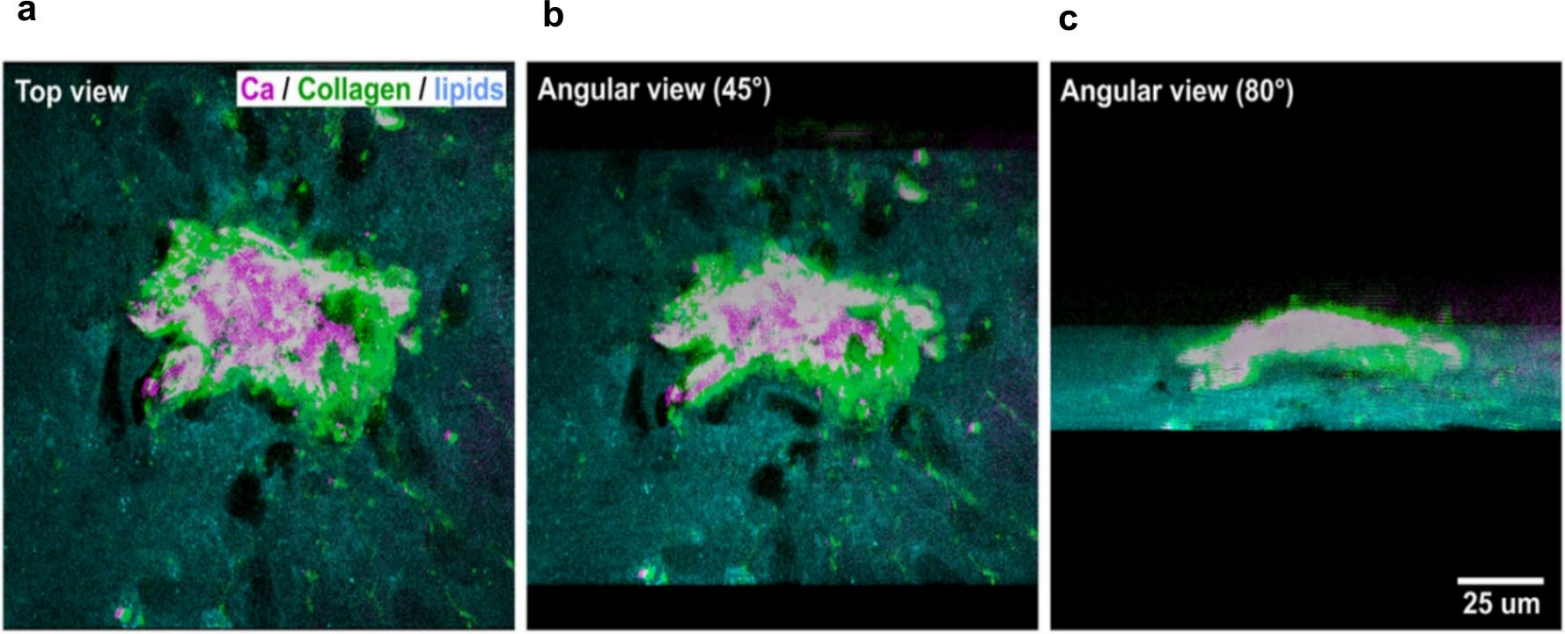
Coherent anti-Stokes Raman scattering (CARS) and second harmonic generation (SHG) microscopy three-dimensional imagens from osteodifferentiated human neural crest-derived inferior turbinate stem cells (ITSCs). (**a**) top view, and angular views of (**b**) 45º and (**c**) 80º, revealing the three-dimensionality of ITSC-derived calcium hydroxyapatite deposits (Ca, purple) deposits embedded in collagen (green). Adapted from [79], licensed under Creative Commons Attribution 4.0 International (CC BY 4.0) license

Regarding the recognized promise of several advanced vibrational microspectroscopic methods in the context of stem cell differentiation [32, 35], we highlight an earlier CARS application to inferior turbinate stem cells (ITSC), which are known to exhibit several similarities to MSC [79]. CARS was employed, in tandem with second harmonic generation (SHG) microscopy, to image the molecular events taking place during ITSC osteogenic differentiation. While the expected increase in the ν_s_ $({\text{PO}}_{4}^{3-}$) band at 959 cm^−1^ is readily noted due to HA deposition between days 14 and 21, CARS microscopy was able to image HA deposits with high sensitivity and spatial resolution in 3D maps (Fig. 7a-c) using the lipid band at 2845 cm^−1^ to visualize whole cells, and the 959 cm^−1^ band to identify HA deposits. The latter seemed to clearly co-localize with type-I collagen sites.

Further fundamental knowledge on the biochemistry of MSC osteodifferentiation has been sought to identify stimuli other than the traditional biochemical inductive factors (namely, dexamethasone and *β*-glycerophosphate), believed not to be physiologically relevant in the human organism and to often stimulate co-lineaging, as briefly mentioned before. Towards this end, the osteogenic differentiation of Wharton’s jelly MSC (WJMSC) was investigated using sprayed bioactive and biocompatible calcium phosphate substrates with controlled topography and without the aid of growth factors [80]. This was successfully demonstrated by cell mechanobiology and integrin expression measurements, with FTIR and confocal Raman spectroscopy (in the 600–1800 cm^−1^ region), mainly used for the characterization of calcium phosphate phases and their degrees of crystallinity. Furthermore, the bioactivity of calcium phosphate substrates was related to their ability to precipitate extracellular Ca and phosphate ions, and indeed substrate incubation in cell medium led to poor crystalline HA (bands at 1115 cm^−1^ in FTIR, and at 962 cm^−1^ in Raman), and adsorption of media proteins and lipids. In FTIR, this was shown by bands at 1640 and 1535 cm^−1^ for Amides I and II, and at 1460–1380 cm^−1^ for CH_2_ and CH_3_ lipid acyl bands. In Raman, signals at 1595 and 1377 cm^−1^ (interpreted as Amides I and III) and at 733 and 1470 cm^−1^ (lipids ν(C–C) and in-plane (CH_2_) deformation) were equally informative.

In addition, a Raman study of MSC was particularly focused on the metabolism of OCN, one of the non-collagenous ECM proteins, used as a late marker of osteogenic differentiation [81]. Raman spectra were obtained from 10 different locations in the samples, normalized to the quartz coverslips 1050 cm^−1^ band due to its stable intensity. Importantly, the authors considered (and discarded) other bands for normalization, namely those of the Amide I and Phe (which depended on collagen and non-collagenous proteins) or the matrix CH_2_ wag 1449 cm^−1^ (which was observed not to remain constant). The spectra were used to monitor the distribution and dynamics of mineral species during osteogenic differentiation, in control and OCN-knocked down samples. Spectral changes revealed the presence of DCPD (985 cm^−1^), OCP (957 cm^−1^), *β-*TCP (970 cm^−1^) and HA (960 cm^−1^), with signals emerging sequentially in time, although at a slower rate (together with slower MTMR evolution) when OCN expression was low. Knocked down OCN also resulted in the down regulation of several osteogenic markers such as the runt-related transcription factor 2 (RUNX2), ALP, osteonectin and type-I collagen, while the transcription factor osterix was found to be upregulated [81]. It was, thus, established that OCN seems to influence mineral species regulation in an exquisite manner, warranting further Raman studies, combined with X-ray and scanning electron microscopy, to provide deeper insight into suspected further mineral species and their distribution.

**Fig. 8:**
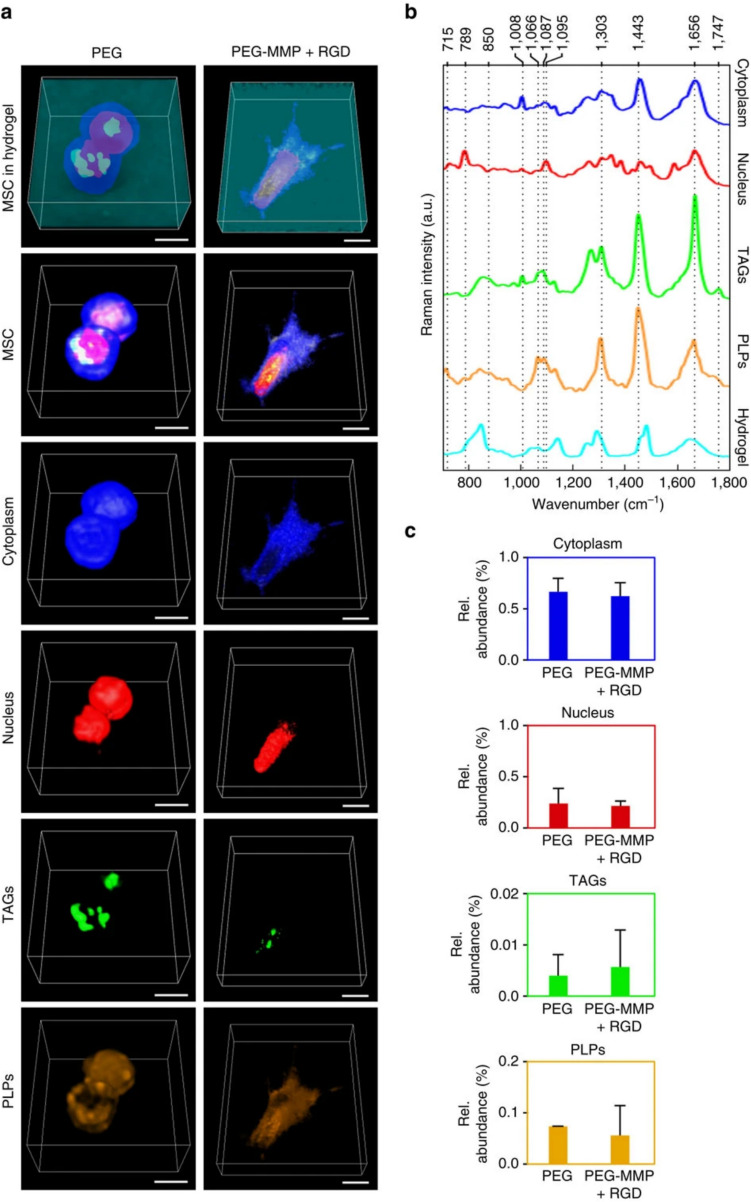
Volumetric quantification of cellular biochemical components in a 3D MSC culture system. Representative 3D reconstructions by qVRI, of the main subcellular components of hMSCs (*n* = 2 cells per hydrogel) in (**a**) a bioactive (PEG-MMP (matrix metalloproteinase) + RGD (arginylglycylaspartic acid)) and a bioinert (PEG) hydrogel, and their corresponding (**b**) endmember Raman spectra from VCA (showing five components); from top to bottom cytoplasm (blue), nucleus (red), triacylglycerols (green), phospholipids (orange) and hydrogel (cyan). (**c**) Bar chart of mean abundance values for each subcellular component. Error bars represent one standard deviation around the mean. Scale bar, 10 mm. Adapted from reference [39] licensed under Creative Commons Attribution 4.0 International (CC BY 4.0) license

Lastly, a mention is warranted to a study of BMSC by quantitative volumetric Raman imaging (qVRI), although not directly investigating MSC osteogenic differentiation. This method enabled exquisite intracellular information to be retrieved on 3D hydrogel culture systems, namely based on bioinert and bioactivated polyethylene glycol (PEG) networks [39]. Based on the use of VCA to “unmix” spectra of different biomolecular components, from hyperspectral datasets collected for multiple z-axis layers, the authors presented a comprehensive set of images unveiling exquisitely fine detail on cell shape, cytoskeleton structures, nuclei, protein clusters, intracellular lipids, membrane lipids (Fig. 8). Offering the possibility of volumetric quantification of these biomolecules in 3D cultures, the promise of this approach to monitor dynamic processes such as differentiation becomes clear.

## Conclusions and Future Perspectives

The developments reported so far on the osteogenic differentiation of MSC through the application of vibrational spectroscopy, in tandem with microscopy, clearly illustrate the importance of these methodologies as label-free, rapid and non-invasive tools to survey single cell behavior (including live cells), over relatively long periods of time (several weeks) and with high spatial detail (*e.g.,* on molecular components distribution). Advances in band assignment and definition of band ratios to assess ECM mineral crystallinity and maturity, as well as collagen formation and maturation, have provided significant insights into the nature and dynamics of molecular events during in vitro fabrication of bone tissue. More recent interests have begun to address the identification/role of minor ECM components such as particular marker proteins, proteoglycans or glucosaminoglycans, as well as lipid moieties either in the form of cytosolic lipid droplets or as membrane components. Several new questions have also arisen *e.g.,* including the role of small molecules and extracellular vesicles in the biology of MSC osteodifferentiation. These questions are linked to the issues of MSC heterogeneity and the unequivocal need for efficient identification and guidance of cells towards pure-lineage outcomes and characteristics close to those of native bone tissue. The important issue of MSC heterogeneity in osteodifferentiation has begun to be addressed by vibrational spectroscopy but still calls for further studies towards the rapid identification of best performing donors/batches for efficient scale-up outcomes. In this respect, the continuous development of higher signal to noise vibrational spectroscopic tools, with higher spatial resolution to unambiguously enable subcellular compartmentalization characterization at the molecular levels, will be important steps forward. Techniques based on intensity-enhanced confocal Raman microspectroscopy *e.g.,* involving CARS and SERS, as well as SR-FTIR and FPA-FTIR spectroscopies, are pushing the spatial resolution and sensitivity of vibrational analysis to new subcellular limits. This will certainly lead to applications extending well into MSC cultures in complex 3D biomaterials of relevance, naturally provided that the biomaterials are well characterized in terms of their degree of transparency to IR and Raman radiation.

We foresee that some analytical issues will also require future careful consideration, namely the definition of validated standard operating procedures (SOP) across laboratories to ensure comparability, not only regarding cellular characteristics and sampling (*e.g.,* cell confluence, actual status of live cells when relevant) but also in relation to the nature and quality of experimental spectroscopic data. These needs increasingly call for SOP related to protocols of data pre-processing (namely regarding normalization and scaling procedures), handling and model validation, particularly if multivariate analysis and further machine learning tools are employed. The promise of more elaborate artificial intelligence methodologies in the interpretation of complex spectral profiles opens a clear additional avenue in the present context. However, albeit their promise, these increasingly sophisticated data mining methods will always rely on efficient SOP regarding the previous steps of sampling and data processing, as these are typically expected to be largely prone to user-dependent artefacts.

Therefore, vibrational microspectroscopy applicability in building classification and prediction models for the rapid assessment of cell outcomes, such as differentiation, will necessarily require an important investment in model validation and strict robustness assessment. The emerging powerful methods of machine learning (and eventually more complex AI methods) will also foster the combination of vibrational spectroscopic data with other data domains (biochemical, metabolomic, genetic, proteomic, physical data and even cell donor metadata), so that stronger predictive multidisciplinary markers of MSC osteogenic differentiation may be envisaged and eventually translated to in vivo settings and clinical practice.

## Data Availability

Not applicable.
